# Bioinspired Fabrication of one dimensional graphene fiber with collection of droplets application

**DOI:** 10.1038/s41598-017-12238-1

**Published:** 2017-09-21

**Authors:** Yun-yun Song, Yan Liu, Hao-bo Jiang, Shu-yi Li, Cigdem Kaya, Thomas Stegmaier, Zhi-wu Han, Lu-quan Ren

**Affiliations:** 1 Key Laboratory of Bionic Engineering (Ministry of Education), Jilin University, Changchun, 130022 P.R. China; 2 German Institutes of Textile and Fiber Research Denkendorf, Denkendorf, Germany

## Abstract

We designed a kind of smart bioinspired fiber with multi-gradient and multi-scale spindle knots by combining polydimethylsiloxane (PDMS) and graphene oxide (GO). Multilayered graphene structures can produce obvious wettability change after laser etching due to increased roughness. We demonstrate that the cooperation between curvature and the controllable wettability play an important role in water gathering, which regulate effectively the motion of tiny water droplets. In addition, due to the effective cooperation of multi-gradient and multi-scale hydrophilic spindle knots, the length of the three-phase contact line (TCL) can be longer, which makes a great contribution to the improvement of collecting efficiency and water-hanging ability. This study offers a novel insight into the design of smart materials that may control the transport of tiny drops reversibly in directions, which could potentially be extended to the realms of in microfluidics, fog harvesting filtration and condensers designs, and further increase water collection efficiency and hanging ability.

## Introduction

In spite of fog collection has been implemented for centuries, its water output is greatly neglected by water conservancy department due to the low efficiency in fog-collection^1^. Fortunately, many biological surfaces, after million years of evolution in nature, possess a capability of controlling water drop behavior. For example, desert beetles use special structure on their back, which is an array of hydrophilic bumps distributed on a super hydrophobic background, to capture water from humid air^2^. Spider silks rely on the cooperation of wettability gradient and Laplace pressure on spindle knot to move tiny water drops^3^. A special microstructure on the back of lizards is capable of directed liquid transport due to capillary water transport mechanism^4^. Especially, spider silks have caught people’s eyes due to their superior fog collection ability^5–8^. It is found that the wettability gradient and Laplace pressure on the wetted capture spider silks are cooperated to move tiny water drops^9^, which opens new routes for the development of functional fibers. Zheng *et al*. successfully fabricated multi-gradient and multi-scale spindle knots on fibers, and revealed multi-scale spindle knots collect together more fog than single-scale spindle knots due to longer three-phase contact line (TCL)^10^. Jiang *et al*. fabricated a novel multi-scale structured membrane with radiate distribution of beaded fibers for oriented water transport^11^. In addition, bioinspired tilt-angle fabricated structure on gradient fibers was fabricated to enable micro-drops fast transport in a long-distance^12^. However, most of studies only have focused on water drop unidirectional transport capacity, and cannot control the transport of tiny drops reversibly in directions. Controlling the transport of tiny drops reversibly occupies an important position in many engineering applications, such as microfluidics design^13^, fog harvesting^14,^filtration^15^, and condensers^16^. For spider silk, the motion of droplet with controllable direction (“toward” or “away from” the knot) is related to curvature, chemical composition, and roughness gradients on the fiber surfaces^3^. Although people have tried to fabricate smart materials with various responses, including optics^17^, temperature^18^, and surface roughness^3^, to control the wettability of the surface, and then control the moving direction of droplets, most of the traditional materials are limited by complex process, high cost and single intelligent response. Specifically, it is far from satisfying the demand of the directional transport of water droplets and the intelligent control of water droplets in the microfluidic chip, specialized liquid handling, heat exchangers, and self-powering actuators for traditional materials.

Considerable research efforts have been devoted to graphene and graphene-related materials, since they have exhibited many excellent properties, including optical reflectance and transmittance^19,20^, high mechanical strength^21^, better conductivity^22^, flexibility^23^, chemical stability^24^, and biocompatibility^25^. However, much work so far has focused on the electronic characters, and in fact the surface wettability control of graphene has attracted much attention. Benefitting from its native structure and chemical properties, graphene shows great potential for the realization of wettability control. For example, Koratkar *et al*. used different solvent technique to control the wettability of a solid surface by deposition of a graphene film on the surface^26^. The wettability of the vertical graphene nanosheets could be controlled from hydrophobic to hydrophilic nature by introducing defects^27^ or chemical modification method^28,29^. Additionally, Nam *et al*. reported that substrate doping-induced charge carrier density modulation leads to the tunable wettability and adhesion of graphene^30^. However, up to now, the relative reports of graphene in fog collection and regulation of tiny water droplets motion are scarce. Thus, based on the wettability regulation of graphene surface, we realize the control of motion of tiny water droplets. Meanwhile, artificial multi-scale bioinspired fibers can be fabricated to enhance the hanging ability and fog collection efficiency.

**Figure 1:**
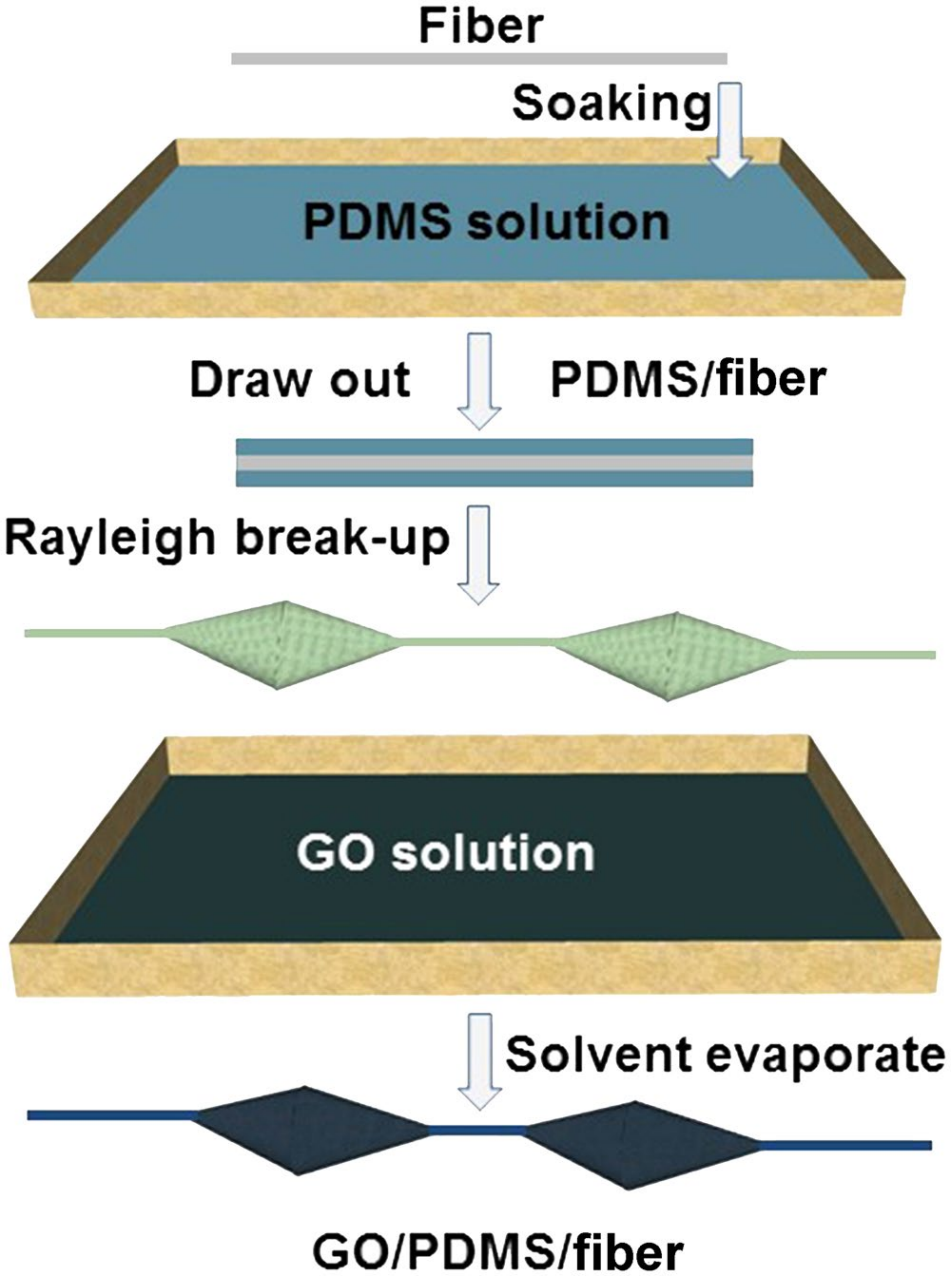
Scheme of fabrication of a bioinspired fiber with roughness gradient. When the fiber is drawn out from the PDMS solution at a high rate, a thin solution film is formed on the fiber, and subsequently it breaks up into regularly-distributed primary spindle-knots. After immersed in graphene solution, graphene film is attached on the surface of PDMS spindle-knots.

In this paper, we fabricated a series of the bioinspired fibers combing PDMS and graphene to regulate the droplet motion by wettability regulation and investigate the influence of geometry on hanging-drop ability and collecting water efficiency. A multi-level spindle knot was formed via twice dip-coating technique (as illustrated in Fig. 1)^3^, and thus the driving force for tiny droplets could be enhanced to the spindle-knots of the fiber. Multilayered graphene structures can produce obvious wettability change after laser etching. We demonstrate that the cooperation between curvature and the gradient wettability direction play an important role in water gathering and transport, which regulate effectively the motion of tiny water droplets. This study offers a novel insight into the design of smart materials that may control the transport of tiny drops reversibly in directions, and further increase water collection efficiency and hanging ability.

## Results and Discussion

### Morphology and geometrical structure of bioinspired fiber

**Figure 2:**
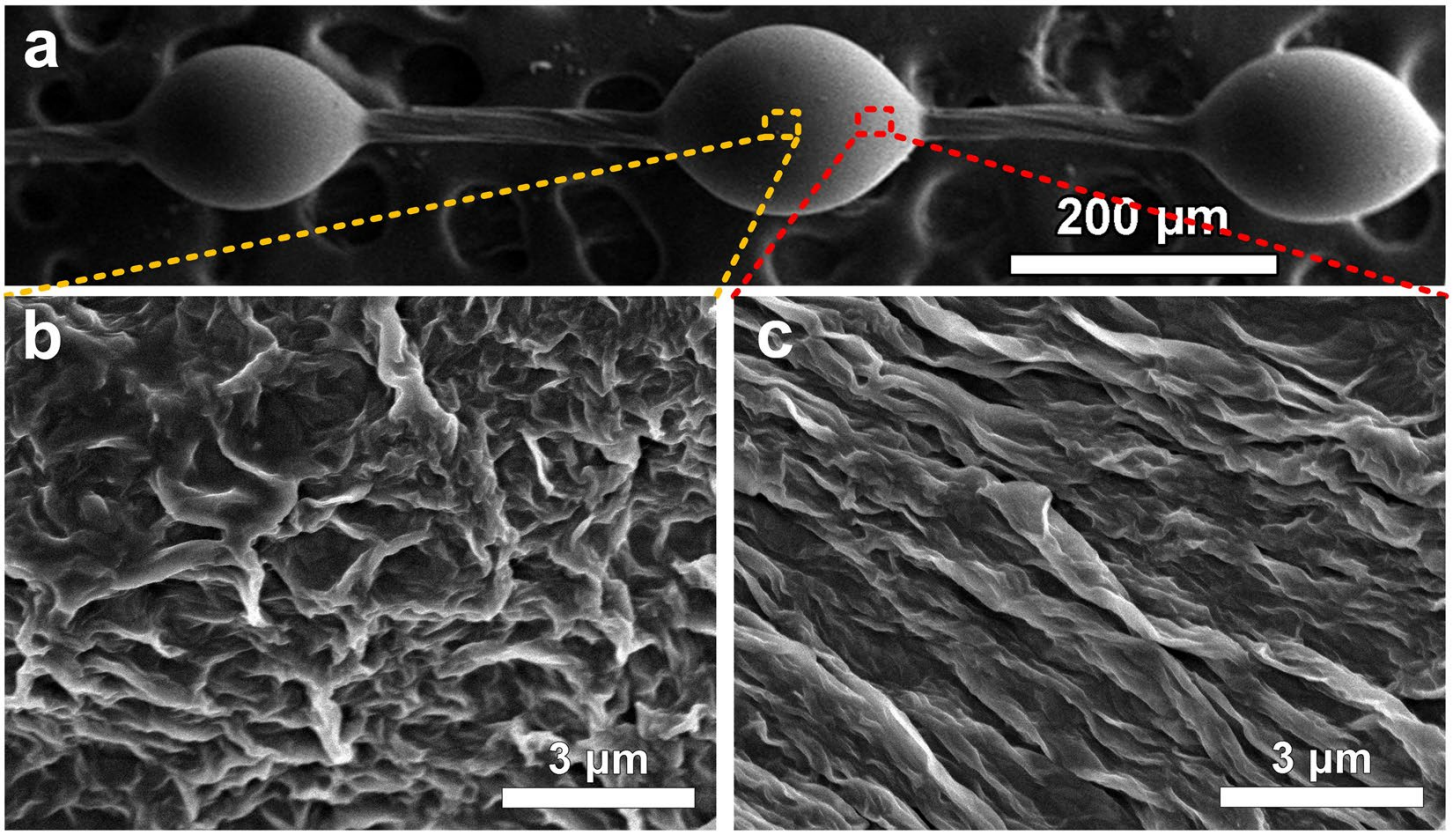
Structural features of bioinspired fiber with GP spindle-knot. SEM images show a single geometric spindle knot in (**a**), and the roughness gradient that forms from the center region (**b**) to the side region of a spindle knot (near the connecting part), accompanying the anisotropic distribution due to the structure extension (**c**).

The microstructures of multilevel graphene on GO/PDMS (GP) spindle-knot are illustrated in Fig. 2. As for the microscale structure feature, Fig. 2a–c show the scanning electron microscopy (SEM) images of a graphene bioinspired fiber. It is worth noting that, the gradient roughness is formed along spindle knot from the center region (Fig. 2b) to the side region (Fig. 2c), accompanied with anisotropic layered distribution due to the structure extending. Thus, water drops can be driven by the surface energy gradient arising from differences of roughness^16^. In addition, the spindle-shaped geometry will generate a difference in Laplace pressure^3^, which will cooperatively favor this directional movement of drops toward the spindle knots. Furthermore, the coalescence energy of drops merging along the spindle knot is another driving force for drop’s directional movement^31^. The process of coalescence caused water drop deformation and reduced the area of the solid–liquid interface, which was similar to the process of droplet colliding with the ground and bouncing into air. The surface energy released in the coalescence process drived water drop directional motion to the center of spindle knot. Then the surface energy released in the coalescence process is given by Equation 1 in supporting information. However, hydrophilicity contributes to easy exfoliation of GO on the surface of GP spindle knot, and decreases fog collecting efficiency. Thus, to overcome these difficulties, the bioinspired fibers with GP knots were reduced by hydrazine hydrate, with the aim to eliminate surface oxygen functional groups and reduce catalytic activity.

### Component of bioinspired fiber

**Figure 3:**
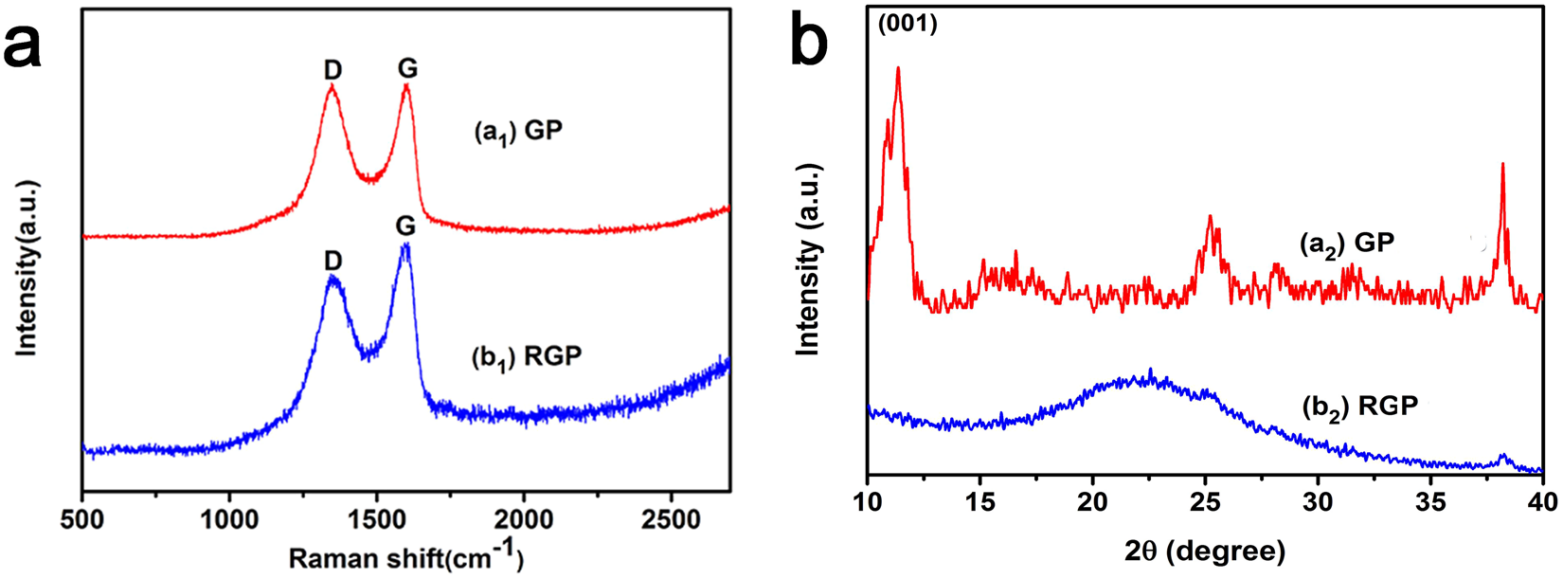
(**a**) Raman spectra of (a_1_) GP spindle-knot, (b_1_) RGP spindle-knot. (**b**) XRD patterns for (a_2_) GP spindle-knot, (b_2_) RGP spindle-knot.

In order to prove that GO has been reduce by chemical method, we measured the Raman spectra and XRD patterns of GP spindle-knot and RGP spindle-knot as shown in Fig. 3. Compared with GP spindle-knot, the intensity of D band relative to G band of RGP (reduced GP) spindle-knot is significantly lower than that of graphite oxide (Fig. 3a_1_), suggesting that GO has been converted into RGO (reduced GO)^32^. Meanwhile, the XRD diffraction patterns of GP spindle-knot and RGO/PDMS (RGP) spindle-knot are displayed in Fig. 3b. GO had a typical sharp peak at 10.8° (Fig. 3a_2_), consistent with the previously reported results^33^. Figure 3b_2_ endowed with a pronounced but quite diffused peak centered at 24.4°, belonging to the joint actions of the PDMS and RGO plates^34^. These results indicate that chemical reduction process at 95 °C was sufficient to reduce GO completely and allowed the functional graphene to be well dispersed and attached to PDMS spindle knot.

**Figure 4:**
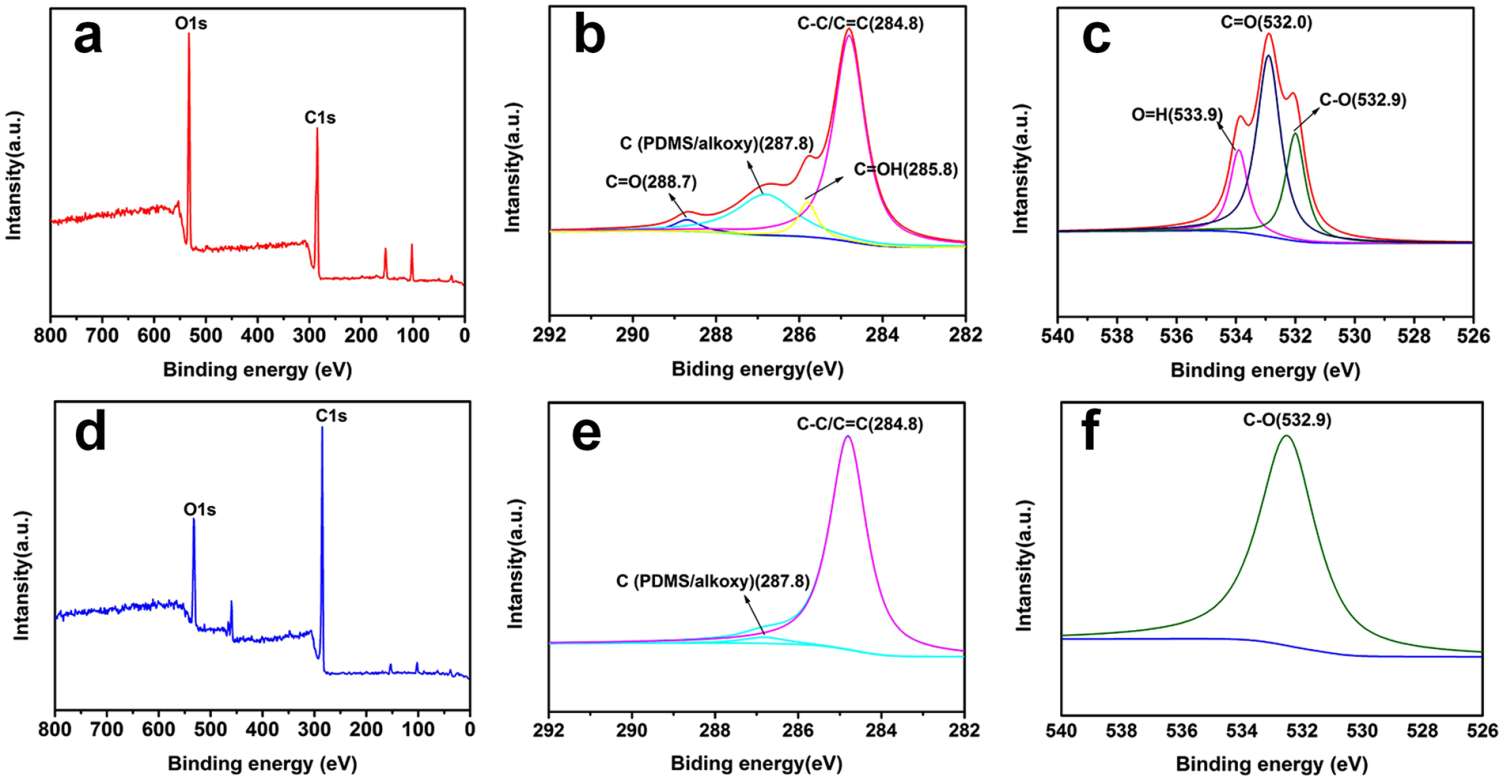
XPS survey scans of (**a**) GP and (**d**) RGP spindle-knot. C1s XPS spectra of (**b**) GP and (**e**) RGP spindle-knot, O1s XPS spectra of (**c**) GP and (**f**) RGP spindle-knot.

XPS was an efficient tool to confirm the chemical compositions on the surfaces of GP spindle-knot and RGP spindle-knot. Figure 4a and d illustrate the XPS survey scans of GP and RGP spindle-knot. When compared with GO in Fig. 4a, the intensity of C1s peak in Fig. 4d shows significant increase, suggesting the formation of RGP spindle-knot. In addition, as shown in Fig. 4b, there were four typical peaks at 284.8, 285.8, 286.8 and 288.7 eV, ascribed to unoxidized graphite carbon (C**-**C/C=C), C-OH, C (PDMS/alkoxy) and C-O groups, respectively^35^. Interestingly, for RGP spindle-knot (Fig. 4e), the C1s peak, in addition to unoxidized graphite carbon and residual C (PDMS/alkoxy), most of the oxygen-containing functional groups have been removed after reduction process. Compared with O1s XPS spectra of GP spindle knot in Fig. 4c, the O1s XPS spectra of RGP spindle-knot in Fig. 4f shows obvious decrease in oxygen functional groups (such as O-H, C=O). This component analysis further verified GO attached on PDMS spindle knot has been reduced.

### Fog collection of bioinspired fiber

**Figure 5:**
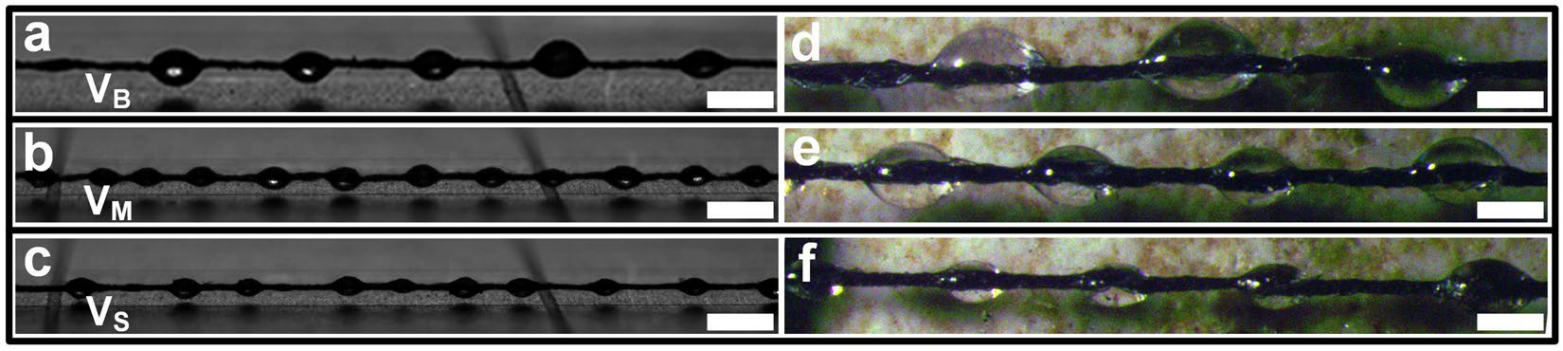
(**a**–**c**) and (**d**–**f**) Optical and Stereo microscope images of PDMS spindle knot on fiber surface with different size spindle-knots respectively (**a**–**c** scale bar: 60 μm, d-f scale bar: 200 μm). (Spindle knots with maximum volume: V_B_, spindle knots with middle volume: V_M_, spindle knots with minimum volume: V_S_).

To prove the effects of geometrical structure of bioinspired fiber on hanging ability and fog collecting efficiency, a series of the bioinspired fibers with different size spindle knots were fabricated by controlling drawing rates. Figure 5a-c illustrate optical microscope images of bioinspired fiber with different size PDMS spindle-knots respectively, which are named with V_B_ (big spindle knot: 0.38 nL), V_M_ (medium spindle knot: 0.14 nL) and V_S_ (small spindle knot: 0.04 nL)_._ For high viscosity PDMS solution, with the increase of drawing velocity, the size of spindle knot decreases accordingly. Thus, we obtained bioinspired fibers with different size spindle-knots by controlling drawing-out velocity. Meanwhile, to observe surface topography of spindle knots, Figure 5d–f refer to stereo microscope images of V_B_, V_M_ and V_S_. Apparently, the width and height of the V_B_ spindle knots are even greater than V_M_ and V_S_, which indicates that drawing velocity has a significant influence on the size of the spindle knot. Additionally, we fabricated bioinspired fibers with different size GP spindle-knots, and RGP spindle-knots to evaluate the size of hump-on-string and graphene on the hanging capacity and fog collection efficiency. The microstructures of multilevel graphene on GP spindle-knot are nearly the same with RGP spindle-knot in spite of different size spindle-knot (Figs S1 and S2 illustrated the microstructures of V_B_ GP spindle-knot and V_M_ RGP spindle-knot in supporting information).

**Figure 6:**
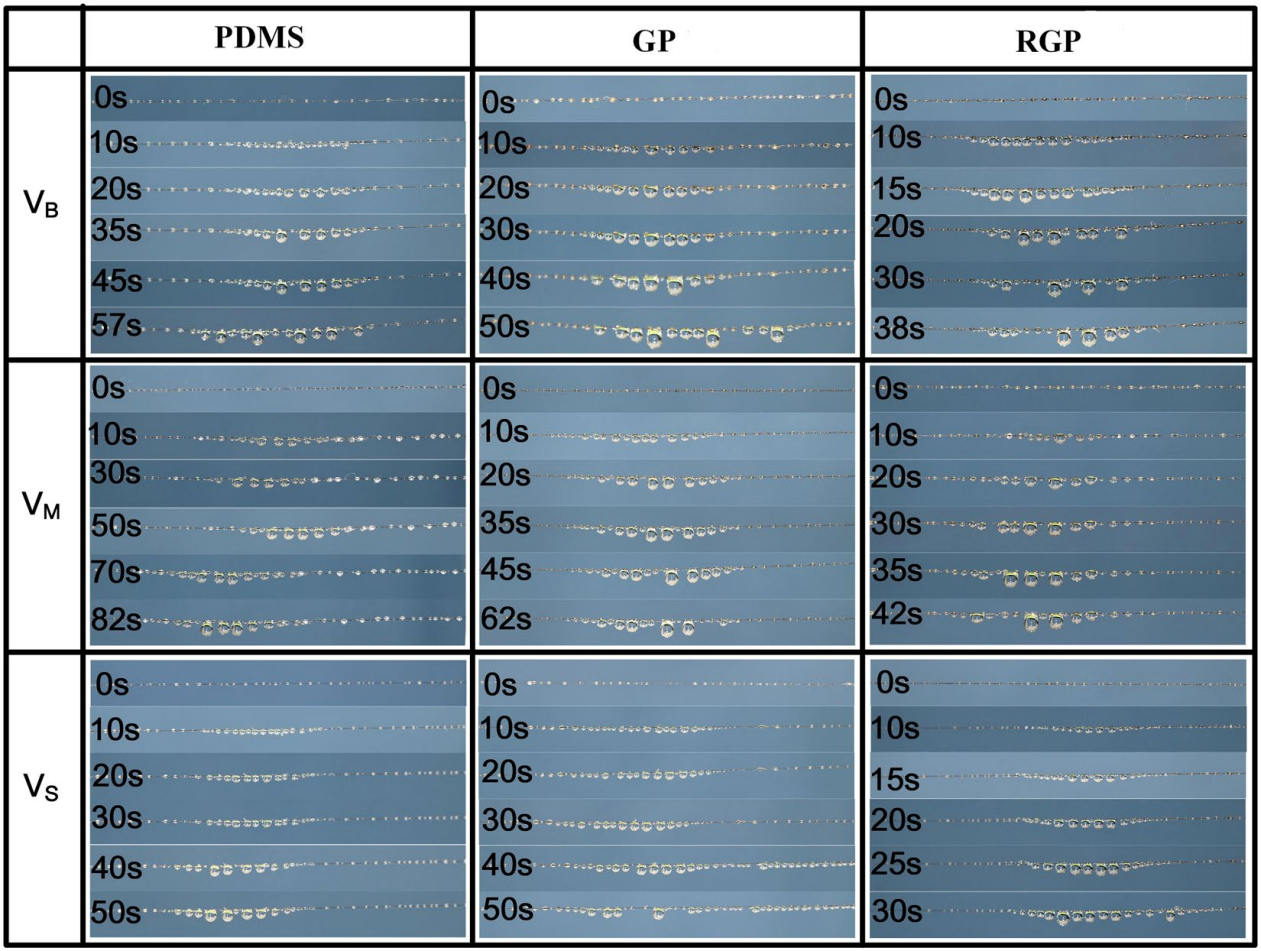
Observation and illustration of water collection on multi-scale spindle knots of bioinspired fiber with different size spindle-knots and graphene nano-structure.

In order to clearly observe the collection behavior of water drops, bioinspired fibers were placed between two holders with humidity 90–95% via controlling a humidifier. The numerous tiny water drops can be collected on the bioinspired fiber. The collection behavior of water drops on the bioinspired fiber was observed by a single-lens reflex camera with time scale. Figure 6 illustrates the fog collecting process of bioinspired fibers with different size PDMS, GP, and RGP spindles including V_B_, V_M_ and V_S_. With continuous fog impact, there are several water drops formed on the spindle knots of bioinspired fibers. The cooperation of Laplace pressure and wettability gradient force contributes to a sequence of drop coalescence. Meanwhile, the coalescence energy drives water drops moving to the center of spindle knot, and accelerates fog collection velocity. Firstly, to compare the hanging water ability of different size spindle knot, the collection process of PDMS spindle knot were analyzed in detail. As time scale goes, the drops grow continuously due to simultaneous condensation of fog droplets in spite of V_B_, V_M_ and V_S_. However, the maximal water-hanging volume of bioinspired fiber with V_B_ spindle knot obviously exceeds V_M_ and V_S_. For bioinspired fiber, Jiang *et al*. used L = 2 m + πh (denoting m as original periodicity length of spindle knots, h as the original height of spindle knots) to estimate the length of TCL^36^. Figure S3 shows the simulated fog collection process of bioinspired fiber with different size spindle-knots. Initially, it is observed that water drops are collected on every spindle knot of bioinspired fiber and grow gradually. Then the water drop is rooted on the two spindle knots during coalescence of two water drops. Finally, the gravity overcomes the surface force and a water drop detaches from two spindle knots. By comparing the TCL at threshold conditions of V_B_, V_M_ and V_S_, it was found that V_B_ owns the longest TCL due to greater width and height of spindle knot, which provides surface force to overcome gravity, thus resulting in the formation of big hanging water volume. Furthermore, the second possible driving force for directional water drop movement arises from the spindle-shaped geometry of the knots, which will generate a difference in Laplace pressure. As illustrated in Fig. S4, shape of the spindle-knot generates a difference in Laplace pressure difference (*ΔP*) from the high-curvature region (joint) to the low-curvature region. Meanwhile, ΔP was positive correlative with the half apex-angle of the spindle-knot β as shown in Equation 2 in supporting information. With the increase of spindle-knot size, the higher apex-angle of the V_B_ spindle-knot drives the water drop toward the center of the spindle knot quickly (β is the half apex-angle of the spindle-knot), thus resulting in the quick fog coherence and enhancing fog collection efficiency. Besides, the center region of spindle-knot is composed of highly random multilevel structures, while the side region is composed of relatively aligned multilevel structures. Therefore, the center of spindle-knot has a higher roughness than side region, which gives rise to a driving force to move the droplet towards the spindle-knot, given by Equation 3 in supporting information.

To clear the role of multi-layered graphene in hanging water ability and fog collecting efficiency, the fog collection process of PDMS, GP, and RGP spindle knot were analyzed in detail. Taking the bioinspired fiber with V_B_ spindle knot as an example, before the water drop detaches, it is observed that critical water drops appear on the fiber. It is worth noting that bioinspired fibers with spindle knot covered by graphene have the maximum critical volume. This might be owed to the fact that multi-scale spindle knots have more collecting sites and larger contact length than PDMS spindle knot. Meanwhile, the period time of reaching critical hanging water volume for graphene spindle knot is shorter than PDMS spindle knot, which is because surface energy gradient from multi-scale spindle knots drives the quick condensation of water drop. In addition, hydrophilic property contributes to easy exfoliation of GO on the surface of GP spindle knot and breaks the multi-scale structures. Thus, bioinspired fiber with RGP spindle knot could obtain the same water drop volume as GP spindle knot by shorter time. To further emphasize the role of RGP spindle knots in fog collecting efficiency for bioinspired fiber, we compared the fog collection process of fibers with small RGP spindle knots and no spindle knots, as shown in Videos 1 and 2 in the Supporting information. Obviously, bioinspired fiber with RGP spindle knots could reach hanging water volume in shorter time compared with smooth fiber, due to the surface energy gradient, and the difference in Laplace pressure arising from the conical spindle-knot geometry.

The overall result is that the wettability gradient arising from the multi-scale graphene structures, and the difference in Laplace pressure arising from the conical spindle-knot geometry act cooperatively to drive water coalescence and growth. In addition, the coalescence energy of drops merging along the spindle knot is another enhancing force for drop’s directional movement^31^. The surface energy released in the coalescence process drived water drop directional move to the center of spindle knot. Thus, bioinspired fiber with V_B_ RGP spindle knots has the maximum hanging water volume considering of these points of view.

**Figure 7:**
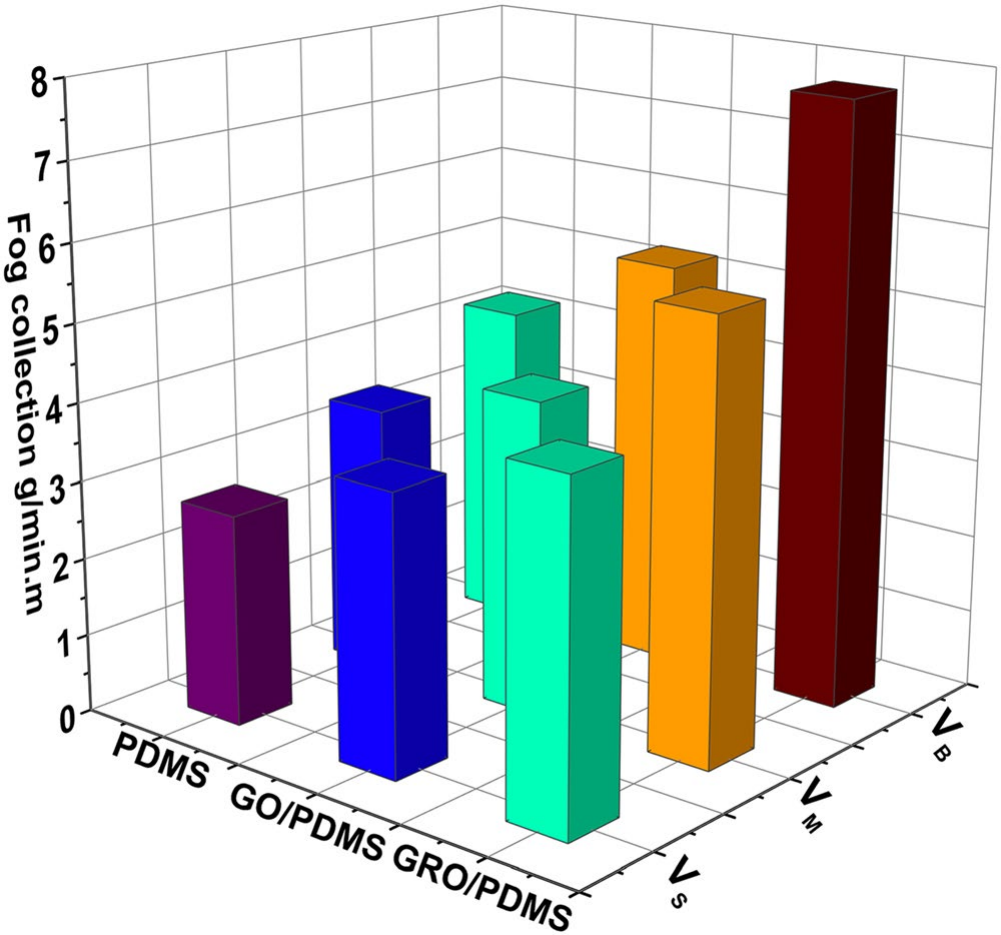
Comparison water collection abilities of bioinspired fiber with different size spindle-knots (V_B_, V_M_, V_S_) and graphene nano-structure (the silimar water collecting rates were expressed in same color).

To examine the fog harvesting efficiency of the series of bioinspired fibers, a quantitative analysis on average water collection rate on different bioinspired fibers with different size PDMS spindle knots, GP spindle, and RGP spindle knot, respectively, is shown in Fig. 7. By comparing the fog collection efficiency, the maximal velocity obtained by V_B_ RGP spindle knot of bioinspired fiber achieves 7.7420 g/min/m, which has a larger hanging drop ability and higher efficiency in water collection than others, because of larger Laplace pressure, width and height for V_B_ spindle knot. Such multi-scale spindle knots increases the length of TCL evidently, which induces a stronger water-hanging ability. Compared with RGP spindle knot, hydrophilicity contributes to easy exfoliation of GO on the surface of GP spindle knot and breaks the multi-scale structures. Thus, bioinspired fiber with GP spindle knot has relatively lower water collection efficiency. In addition, we measured the WCA of PDMS, GP, and RGP spindle knot, respectively, as shown in Fig. S5. The PDMS spindle knot presents a hydrophobicity, which hinders the capture of fog droplets. In contrast, the hydrophilicity of GP promotes it to collect more water in a short time. Though its weak hydrophobic properties, RGO spindle knot collects more water for their stable chemical properties and good binding capacity. The experiment results agree with the previous analysis on water behaviors of different fibers during fog collection.

### Reversible transport of droplets on bioinspired fiber

**Figure 8:**
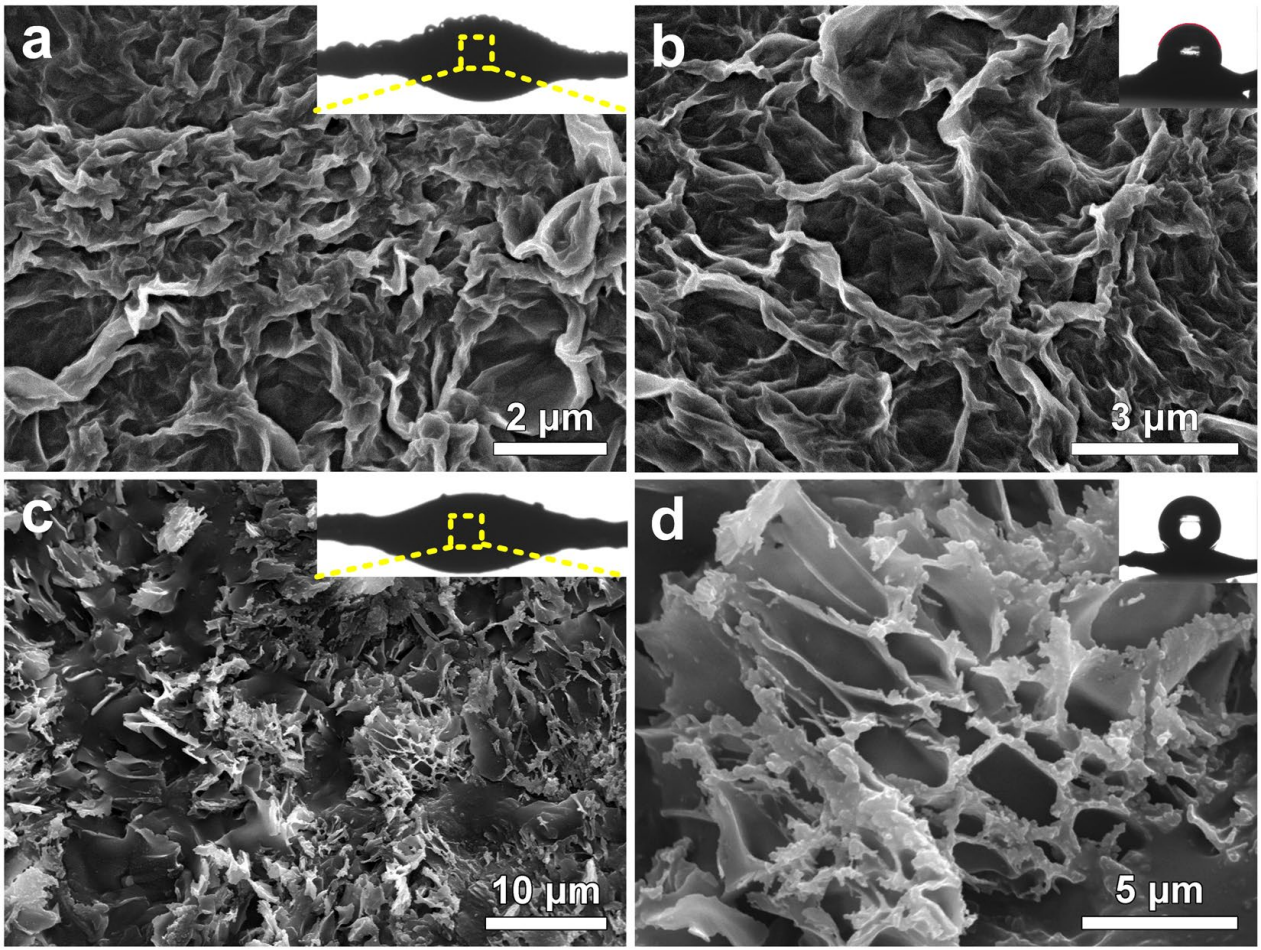
Structural features of bioinspired fiber with RGP spindle-knot. (**a**,**b**) SEM images show the center region of a single geometric RGP spindle-knot in the insets in **(**a). (**c**,**d**) SEM images show the center region of a single geometric LGP spindle knot in the insets in (**c**). The water drop profiles of RGP and LGP spindle knot inset in (**b**) and (**d**) show different wettability characters.

Based on the discussion above, a droplets reversible transport of bioinspired spider silk can be designed by understanding the surface structures of wetting spider silk. To realize the reversible transport of droplets on bioinspired fiber, we changed the surface wettability of RGP spindle knot from hydrophilicity to hydrophobicity by laser etching. That is to etch the center region of spindle knot by laser treatment to change the gradient wettability direction. Thus, the driving force from wettability gradient will overcome Laplace pressure and promote droplets to move away from the center region of spindle knot. The different wetting characters can be illustrated by static contact angle shown in insets of Fig. 8b and d. The change in roughness after laser etching can be characterized using confocal laser scanning microscope (Fig. S5). Obviously, the roughness of laser etched RGP (LGP) spindle-knot is increased from 13.061 to 20.287, which verifies the wettability switching of LGP spindle knot in structures. The structural features of bioinspired fiber with RGP and LGP spindle-knot were shown in Fig. 8. Figure 8a and b show the center region of a single geometric RGP spindle-knot in the insets in a, which still present anisotropic layered distribution. Meanwhile, Fig. 8c and d show the center region of a single geometric LGP spindle knot in the insets in b. Comparably, laser destroyes the multi-layered graphene structure, a jumble of burning marks are left on the surface RGP spindle-knot. Because of the increase of surface roughness and the high temperature reduction of graphene oxide by laser etching, the surface exhibits super hydrophobic characteristics. To further verify the wettability switching of a surface resulting from laser etching, we measured the static contact angle of RGP and LGP spindle knot. The water drop profiles inset in Fig. 8b and d show different wettability characters, indicating laser etching resulting in surface wettability switching of RGP spindle knot from hydrophobicity to super hydrophobicity.

**Figure 9:**
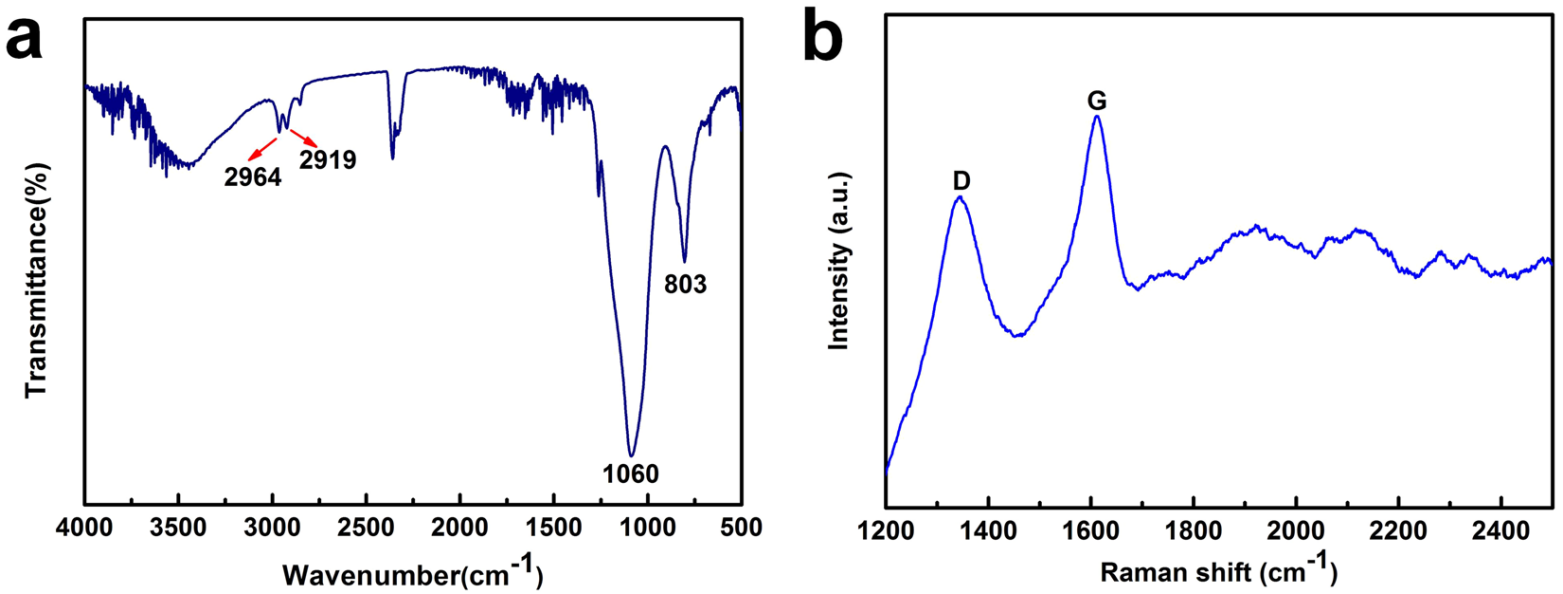
(**a**) FTIR spectrum of the LGP spindle knot. (**b**) Raman spectra of LGP spindle-knot.

In addition, we measured the FTIR spectrum and Raman spectra of LGP spindle-knot in Fig. 9 to consider the component change role in promoting wettability switch. On the one hand, after laser etching, there appeare two weak peaks at wavenumbers of 2964 cm^−1^ and 2919 cm^−1^ (C-H and C–H_2_ stretching in CH_3_). Meanwhile, LGP spindle-knot exhibits distinct IR peaks at wavenumbers of 1060 cm^−1^ (Si–O–Si stretching vibration bond) and 798 cm^−1^ (CH_3_ rocking in Si-CH_3_). These hydrophobic groups contribute to the surface wettability switch. On the other hand, according to the Raman spectra, the intensity of D band relative to G band of LGP spindle-knot is significantly lower than that of GP spindle knot (Fig. 3a_1_), suggesting that graphene has been further reduced due to laser etching.

**Figure 10:**
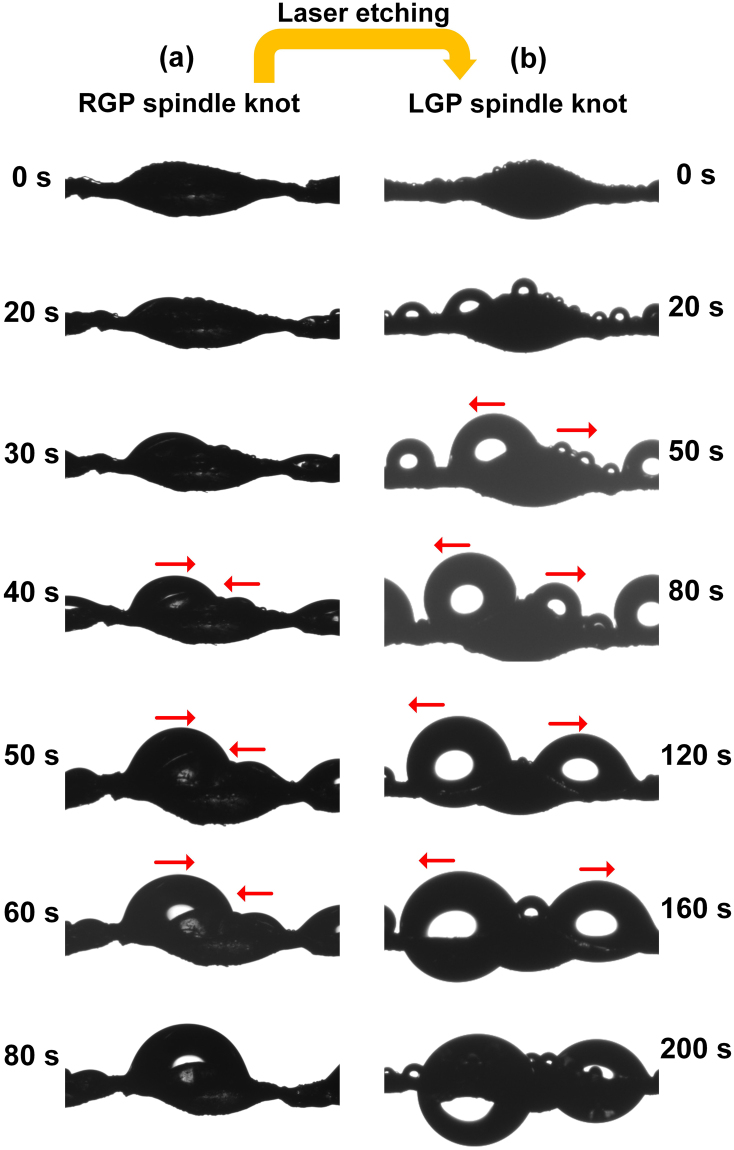
(**a**) On RGP spindle knot, one side of the droplet moves towards the center of the spindle-knot and finally, the droplet coalesces with another droplet at ~80 s. (**b**) On LGP spindle knot, one side of the droplet moves towards the joint and away from the spindle-knot in a period of 0~200 s (indicated with red arrows).

To observe the positive transport (toward the knot) and reversible transport (away from the knot) of tiny drop, the bioinspired fibers with different directional wettability gradient were placed in relative humidity environments provided by a humidifier. The dynamic behavior of droplets on RGP and LGP spindle-knot was recorded using a charge coupled device (CCD) camera at the time scale (Fig. 10). We observed the behavior of droplets on the RGP spindle-knot. Initially, tiny water droplets are gathered randomly on the surface of the spindle-knot, and then, as the droplets grow in a period of 20–80 s, the droplets on joint of bioinspired fiber extend to the spindle knot. In contrast, on LGP spindle-knot, one side of the droplet moves towards the joint and away from the spindle-knot in a period of 0~200 s. Ultimately, the tiny droplets tend to move away from the spindle-knot and grow up on the two sides of spindle knot. For RGP spindle knot, the wettability gradient along spindle knot from the side region to the more wetting center region, and a difference in Laplace pressure resulting from spindle-shaped geometry cooperatively drive water drop toward the spindle knots. Comparably, the center region of LGP spindle-knot demonstrates super hydrophobicity treated by laser etching, but the side region still remain hydrophilicity. Thus, driven by the wettability gradient force, droplets will overcome Laplace pressure and move towards more hydrophilic side region.

## Conclusion

In conclusion, we have successfully fabricated a series of the bioinspired fibers combing PDMS and graphene to investigate the influence of geometry on hanging-drop ability, and collecting water efficiency. Compared to the PDMS spindle knot, RGP spindle knot could induce a much stronger water-hanging ability and higher collecting efficiency. The higher collecting efficiency is attributed to the fact that the multi-scale bioinspired fiber has more collecting sites and a larger contact length to collect more water drops. In addition, multilayered graphene structures can produce obvious wettability change after laser etching due to increased roughness and reduced graphene. Thus, we can regulate effectively the motion of tiny water droplets in directions by controlling the graphene surface wettability. This study is significant for designing smart materials to control droplets transport, and for enhancing fog collection efficiency.

## Methods

### Fabrication of reversible transport bioinspired spider silk

Graphene oxide (GO) was prepared by a modified Hummer’s method from graphite flake^37,38^. The carbon fiber specimens were obtained from silk stocking and the length of the fiber was about 10 cm. Bioinspired fibers with PDMS spindle knots were prepared by immersing carbon fiber specimens in the PDMS solution (10:1, PDMS: crosslinking agent, by weight) and drawing it out by controlling rate to fabricate different size spindle-knots. A cylindrical film of polymer solution was then formed on the fiber surface, and spontaneously broke up into polymer drops along the fiber owing to the Rayleigh instability. After the fiber was dried completely in the ambient environment, periodic polymer spindle knots formed, similar to the geometry of wetted spider silk. To obtain multi-level spindle knots on the fiber, the fiber sample above was further immersed in the GO solution and drawn out at uniform rate. After dry process and chemical reduction, bioinspired fibers with multi-gradient and multi-scale reduced GO/PDMS (RGP) spindle knots were achieved successfully (as illustrated in Fig. 1). To realize the reversible transport (away from the knot) of tiny drop, we changed the surface wettability of GP spindle knot from hydrophilicity to hydrophobicity by laser etching. Thus, the driving force from wettability gradient will overcome Laplace pressure and promote droplets to move away from the center region of spindle knot.

### Characterization

The structures of spindle knots were observed by scanning electron microscope (SEM,Quanta FEG 250, FEI) at 25 kV with gold plating. Surface elemental analysis of the GO/PDMS (GP) and RGP spindle knot was carried out by X-ray powder diffraction (XRD) on a Bruker D8 Focus X-ray diffrac-tometer using Cu K radiation (=0.1542 nm) operated at 40 kV and 40 mA. In addition, X-ray photoelectron spectroscopy (XPS) analysis was performed by an AXIS multifunctional X-ray photoelectron spectrometer (ULTRA DLD, Shimadzu Ltd., Japan at a power of 450 W to verify that GO has been reduced by chemical method. The behavior of water drop was observed via the single-lens reflex camera (Canon 80D, Japan) with time scale.

## Electronic supplementary material

Supplementary Information

Supplementary Video 1

Supplementary Video 2
