# Supplementary material for: Bioinspired Fabrication of one dimensional graphene fiber with collection of droplets application
Source: Sci Rep. 2017 Sep 21;7:12056. doi: 10.1038/s41598-017-12238-1 (PMC5608905; doi:10.1038/s41598-017-12238-1)
Supplement: Supplementary file 1 — Supplementary Information [file 41598_2017_12238_MOESM1_ESM.pdf]

Supporting Information

Bioinspired Fabrication of one dimensional graphene fiber with collection of droplets application

Yunyun Song, Yan Liu*, Haobo Jiang, Shuyi Li, Cigdem Kaya, Thomas Stegmaier, Zhiwu Han, Luquan Ren

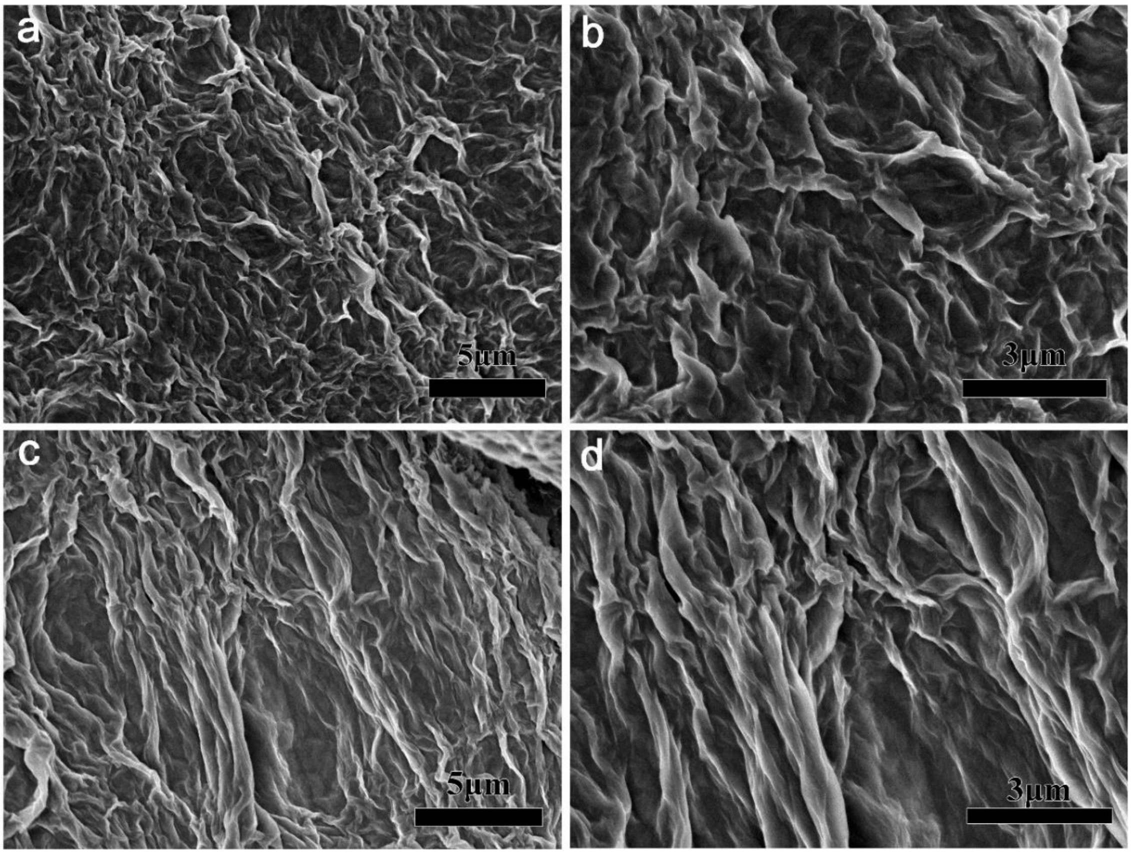

Fig. S1 Structural features of bioinspired fiber with V_B GP spindle-knot. SEM images show the roughness gradient that forms from the center region (a-b) to the side region of a spindle knot (near the connecting part), accompanying the anisotropic distribution due to the structure extension (c-d).

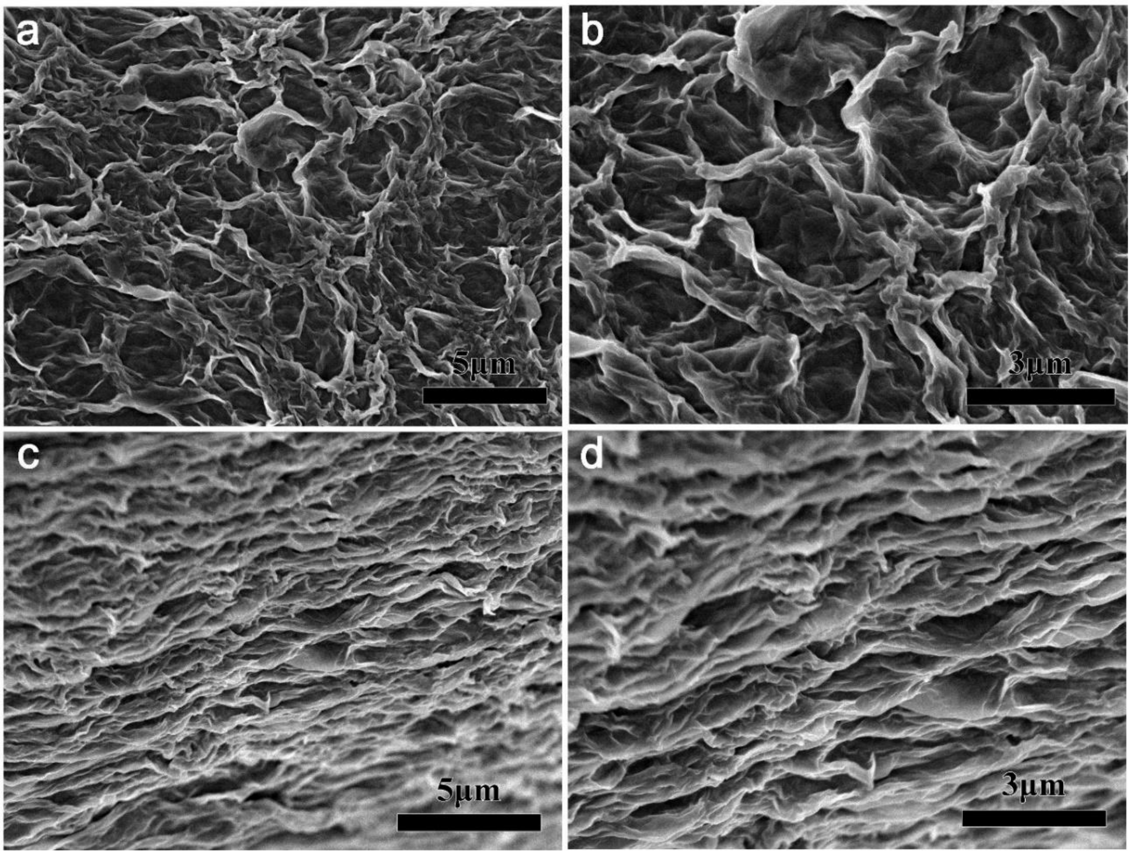

Fig. S2 Structural features of bioinspired fiber with V_M RGP spindle-knot. SEM images show the roughness gradient that forms from the center region (a-b) to the side region of a spindle knot (near the connecting part), accompanying the anisotropic distribution due to the structure extension (c-d).

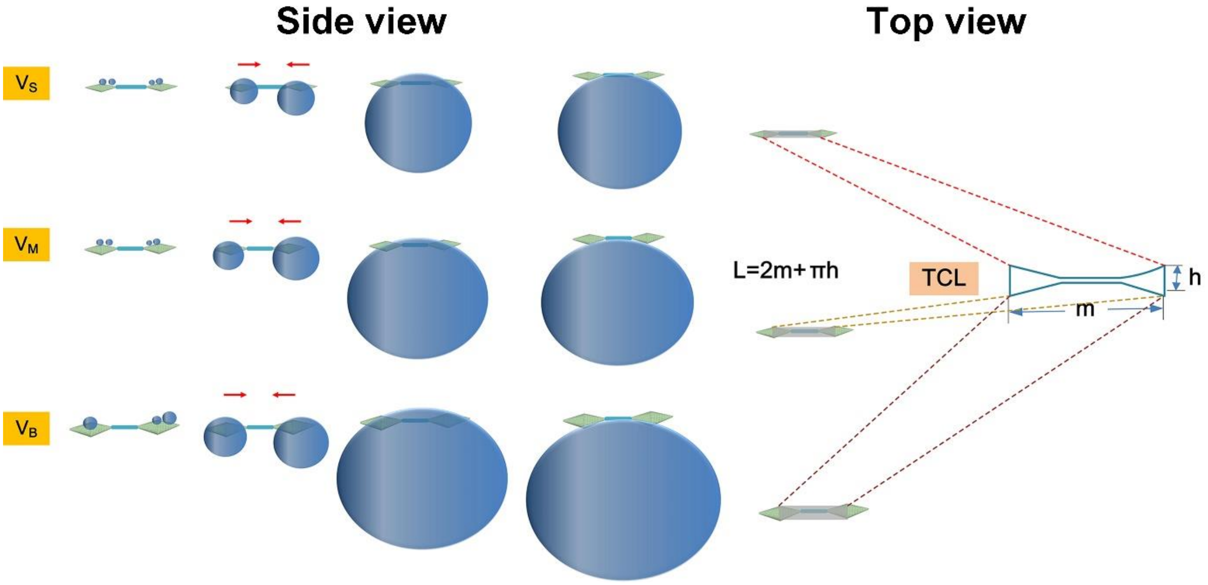

Fig. S3 Illustration of water collection of bioinspired fiber with different size spindle-knots above (including side view and top view; green and blue parts denote bioinspired fiber and water drops, respectively, and blue dashed line denotes the three phase contact line). The length of TCL increases slowly during the water collection process, and when the volume reaches maximal volume, the TCL is composed of one-half-ellipse and two lines and the length (L) of the TCL can be written as $L \approx 2m + \pi h$ (m being the contact length between the fiber and water drop; h being the height of the spindle knot).

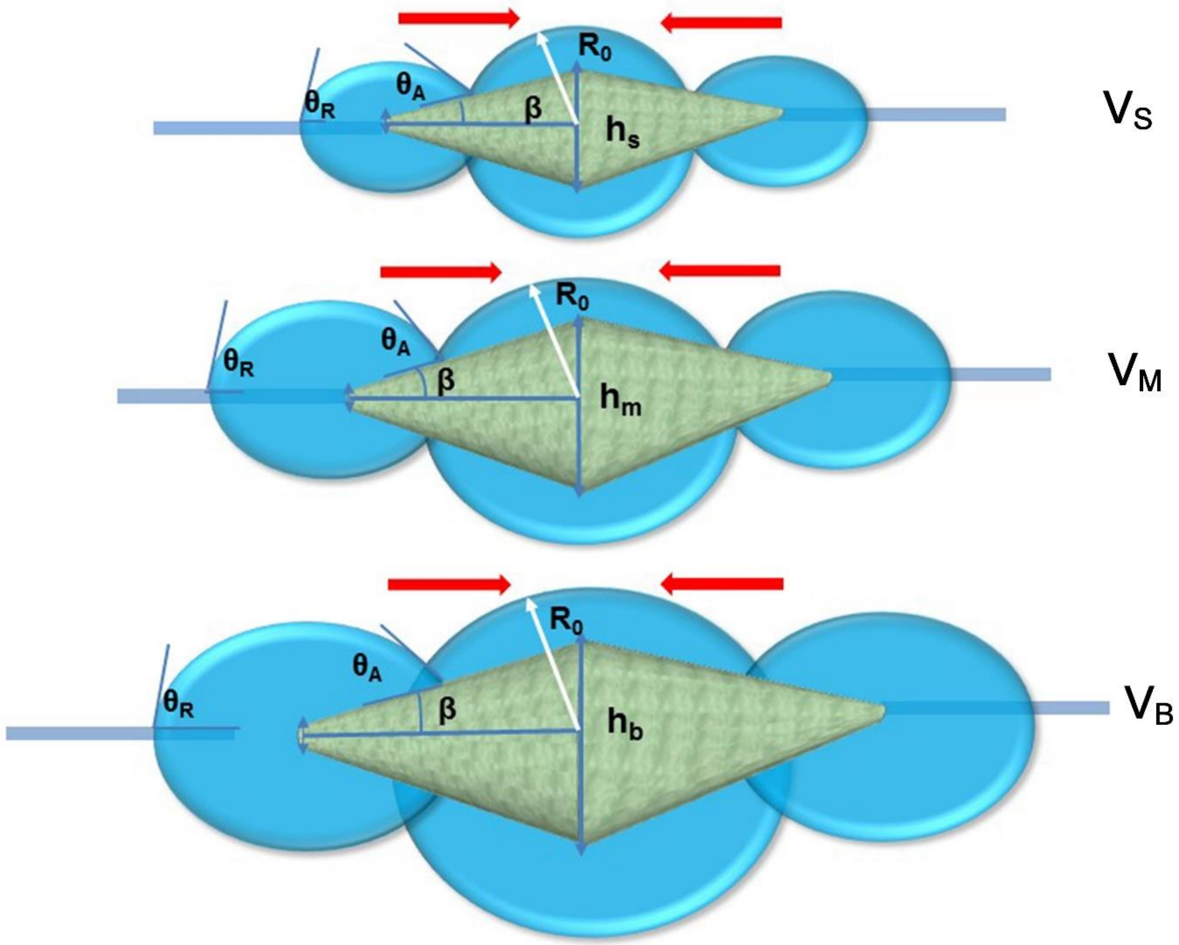

Fig. S4 Mechanism of directional water collection on bioinspired fiber with different size spindle knots. The conical shape of the spindle-knot generates a difference in Laplace pressure from the high-curvature region (joint) to the low-curvature region (spindle-knot). The arrows denote the directions of drop movement. With the increase of spindle-knot size, the higher apex-angle of the spindle-knot drives the water drop toward the center of the spindle knot quickly.

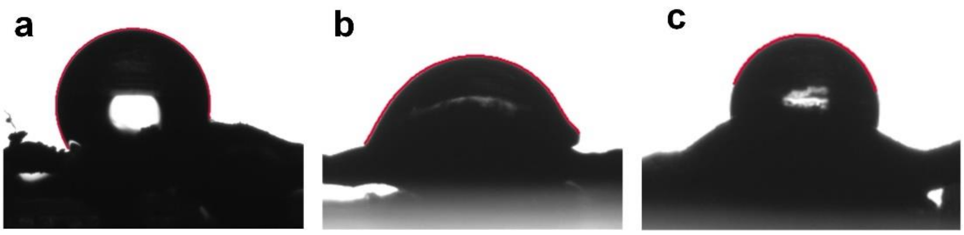

Fig. S5 The water contact angles of PDMS (a), GP (b), and RGP (c) spindle knot.

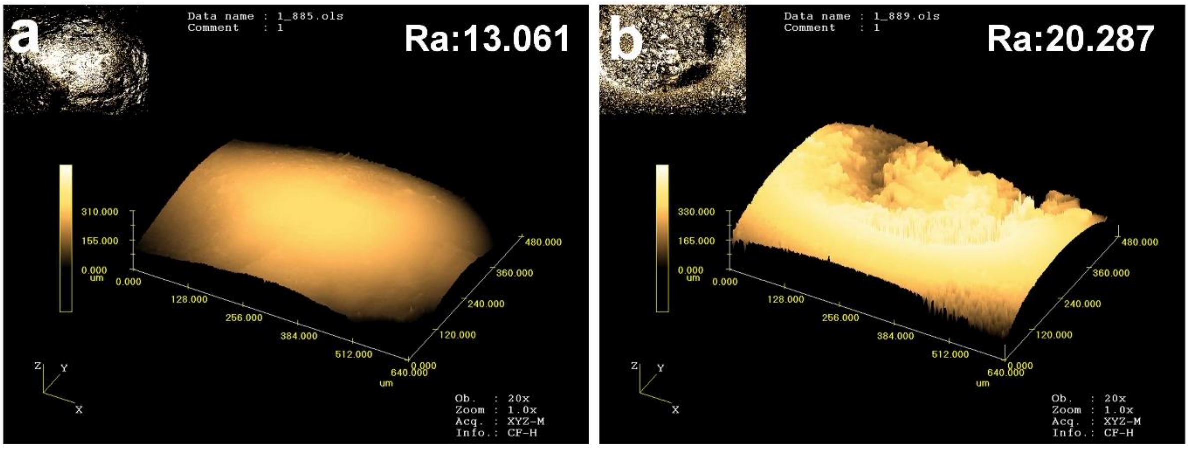

Fig. S6 Laser scanning confocal 3D images of spindle knot before and after laser etching, (a) RGP spindle knot, (b) LGP spindle knot.

Equation 1. In the process of coalescence, the area of the solid−liquid interface reduced is equal to the area of the solid−vapor interface increased. Then the surface energy released in the coalescence process is given by [1]:

$$\Delta E_s = \gamma_{lv}\Delta A_{lv} + \gamma_{sl}\Delta A_{sl} + \gamma_{sv}\Delta A_{sv} = \gamma_{lv}\Delta A_{lv} - (\gamma_{sv} - \gamma_{sl})\Delta A_{sl} \quad (1)$$

Where γ_{lv}, γ_{sl}, and γ_{sv} are the interfacial tension of liquid−vapor, solid−liquid, and solid−vapor, respectively. ΔA_{lv}, ΔA_{sl}, and ΔA_{sv} refer to the variations of area of the liquid−vapor, the solid−liquid, and the solid−vapor interfaces.

Equation 2. Due to conical structure, there exists a Laplace pressure difference (ΔP) acting on a droplet [2]:

$$\Delta \mathrm{P} = -\int_{r_1}^{r_2} \frac{2\gamma}{(r + R_0)} \sin\beta \, dz \qquad (2)$$

Where r is the local radius; R_0 is the droplet radius; β is the half apex-angle of spindle-knot, and z is the integrating variable along the diameter of spindle-knot.

Equation 3. It is worth noting that, the gradient roughness is formed along spindle knot from the center region to the side region, accompanied with anisotropic layered distribution due to

the structure extending, which give rises to a driving force to move the droplet towards the spindle-knot, given by [2]:

$$\mathrm{F} = \int_{L_i}^{L_k} \gamma(\cos\theta_A - \cos\theta_B)dl \qquad (3)$$

Where γ is the surface tension of water; θ_A and θ_R are the advancing and receding angles of droplet on spider silk, respectively; dl is the integrating variable along the length from joint (L_j) to spindle-knot (L_k).

Vedio 1: The fog collection process of fibers with small RGP spindle knots, which could reach hanging water volume and achieve water drop free fall in less than 34 s.

Veddio 2: The fog collection process of smooth fibers. Compared with bioinspired fiber with spindle knots, smooth fiber fog collection velocity was lower, and reached hanging water volume in more than 105 s.

1. Zhou, S., Hao, G., Zhou, X., Jiang, W. & Wang, T. One-pot synthesis of robust superhydrophobic, functionalized graphene/polyurethane sponge for effective continuous oil–water separation. Chem. Eng. J. **302,** 155–162 (2016).
2. Zhang, M. & Zheng, Y. Bioinspired structure materials to control water-collecting properties. Mater. Today **3,** 696-702 (2016).
